# Relationship of inflammatory mediators and sex-related parameters in Jordanian adult men patients with Covid-19

**DOI:** 10.5937/jomb0-35601

**Published:** 2022-10-15

**Authors:** Amneh I. Al-Bashiti, Khaled A. Ahmed, Khalid M. Alqaisi

**Affiliations:** 1 Al-Ahliyya Amman University, Faculty of Allied Medical Sciences, Department of Medical Laboratory Sciences, Amman, Jordan; 2 Al-Ahliyya Amman University, Pharmacological and Diagnostic Research Centre (PDRC), Amman, Jordan; 3 Al-Ahliyya Amman University, Pharmacological and Diagnostic Research Centre (PDRC),Amman, Jordan

**Keywords:** Sars-CoV-2, Covid-19, testosterone, inhibin B, inflammatory mediators, Sars-CoV-2, Covid-19, testosteron, inhibin B, inflamatorni posrednici

## Abstract

**Background:**

Recent epidemiological data suggest that Co - ro navirus disease 2019 (COVID-19) has a gender predisposition, with men being more seriously affected than women. Furthermore, older men accounting for most deaths. Therefore, this study aimed to investigate the serum testosterone, inhibin B, intrleukin-6 (IL-6) and tumor necrosis factor-alpha (TNF-a) levels in different age groups of Jordanian males with SARS-CoV2 infection and to evaluate the correlation of these markers in male patients with COVID-19.

**Methods:**

This study was performed on 157 selected individuals divided into two groups; COVID-19 patients and healthy controls. The participants of each group were further divided into two subgroups based on the age (20-50 years and 51-80 years age groups). The biochemical tests that were performed in this research are testosterone, inhibin-B, TNF-a, and IL-6.

**Results:**

The levels of IL-6 were significantly higher in COVID-19 patients than healthy individuals (7.63 ± 6.30 vs. 5.54 ± 2.10, P=0.006). Similarly, the difference between the levels of TNF-a in the study groups were statistically significant (P=0.001). The mean testosterone levels in COVID-19 patients and healthy controls were 1.53 ± 1.24 and 3.87 ± 1.44, respectively (P<0.001), whereas the mean inhibin B levels in COVID-19 patients (54.29 ± 7.33) were lower than in healthy controls (64.14 ± 37.66) with P = 0.011. TNF-a was significantly and positively correlated with age (r = 0.263, P=0.018) and IL-6 (r = 0.245, P=0.027). Inhibin B had a significant, but negative correlation with TNF-a (r = -0.326, P = 0.003).

**Conclusions:**

It can be concluded that most men seeking medical attention with symptomatic COVID-19 had low testosterone and inhibin B levels with increased both IL-6 and TNF-a, which are independent of age conforming the deleterious effects of SARS-CoV-2 infection on testicular function and immune response induction.

## Abstract (sr)

**Uvod:**

Nedavni epidemiološki podaci ukazuju na to da korona virus 2019 (COVID-19) ima rodnu predispoziciju, pri čemu su muškarci ozbiljnije pogođeni nego žene. Štaviše, stariji muškarci čine većinu umrlih. Stoga je ova studija imala za cilj da ispita nivoe testosterona u serumu, inhibina B, intrleukina-6 (IL-6) i faktora tumorske nekrozealfa (TNF-a) u različitim starosnim grupama jordanskih muškaraca sa infekcijom SARS-CoV2 i da proceni korelacija ovih markera kod muških pacijenata sa COVID-19.

**Metode:**

Ova studija je sprovedena na 157 odabranih pojedinaca podeljenih u dve grupe; pacijenti sa COVID-19 i zdrave kontrole. Učesnici svake grupe su dalje podeljeni u dve podgrupe na osnovu starosne dobi (dobne grupe 20-50 godina i 51-80 godina). Biohemijski testovi koji su urađeni u ovom istraživanju su testosteron, inhibin-B, TNFa i IL-6.

**Rezultati:**

Nivoi IL-6 su bili značajno viši kod pacijenata sa COVID-19 nego kod zdravih osoba (7,63 ± 6,30 naspram 5,54 ± 2,10, P=0,006). Slično, razlika između nivoa TNFa u ispitivanim grupama bila je statistički značajna (P=0,001). Srednji nivoi testosterona kod pacijenata sa COVID-19 i zdrave kontrole bili su 1,53 ± 1,24 i 3,87 ± 1,44, respektivno (P<0,001), dok su srednji nivoi inhibina B kod pacijenata sa COVID-19 (54,29 ± 7,33) bili niži nego kod zdravih kontrola (64,14 ± 37,66) sa P = 0,011. TNF-a je značajno i pozitivno korelirao sa godinama (r = 0,263, P=0,018) i IL-6 (r = 0,245, P=0,027). Inhibin B je imao značajnu, ali negativnu korelaciju sa TNF-a (r = - 0,326, P = 0,003).

**Zaključak:**

Može se zaključiti da je većina muškaraca koji su tražili medicinsku pomoć sa simptomatičnim COVID-19 imala nizak nivo testosterona i inhibina B sa povećanim i IL6 i TNF-a, koji su nezavisni od starosti u skladu sa štetnim efektima SARS-CoV-2 infekcija na funkciju testisa i indukciju imunog odgovora.

## Introduction

Infection with SARS-CoV-2 may lead to activation of macrophages, natural killer (NK) cells, and other immune cells that result in the production of chemokines and cytokines [1]. This dysregulated and hyper-inflammatory response eventually leads to elevated cytokine concentrations, which can be viewed as the primary cause of multiple-organ damage [2]. Increased levels of IL-6 have been linked to death in individuals with severe COVID-19 infection in a retrospective study [3]. Furthermore, Chen and colleagues [4] have found that severe systemic infection is characterized by a combination of variables, including high levels of IL-6, IL-2, IL-10, and TNF-a, low levels of CD4^+^ and CD8^+^ T cells, and lymphopenia. Many of these conditions present in the elderly are accompanied by an inflammatory state expressed by the increasing levels of inflammatory cytokines, including IL-6, TNF-α, and interleukin-1 beta (IL-1β).

There is increasing evidence that men are more likely to die and affected significantly from COVID-19 infection compared to women. For instance, among COVID-19 patients, males are reported to die at twice the rate of females [5]. Studies show that the percentage of infection with SARS-CoV-2 in males is more than females. Moreover, reports from China indicate that men accounted for 60% of COVID-19 positive case patients. SARS-CoV-2 might deteriorate serum testosterone level in infected male patients [6]. Low serum total testosterone levels at baseline have a significant increased risk for the ICU and mortality in patients with COVID-19 [7].

Lower serum testosterone level is a poor prognostic indicator for patients with COVID-19 by deregulating pulmonary protective pathways [8]. Low testosterone levels in males have also a direct correlation with the severity of the disease and a worse outcome in COVID-19 patients [9]. This may explain the higher coronavirus case fatality rate among men compared with women. Malkin et al. [10] have demonstrated that testosterone treatment in hypogonadal men resulted in reductions in TNF-α and IL-1β, and in an increase in IL-10. Cayan and colleague studied the effect of serum total testosterone and its relationship with other laboratory parameters on the prognosis of COVID-19 in infected males in Turkey. Other results showed that low testosterone level at baseline has a significant increased risk for the ICU and mortality in patients with COVID-19 [11]. Furthermore, in their study, Rowland and Bergin [12] have investigated the levels of testosterone for early identification and treatment among 45 German patients at high risk of mortality from COVID-19. They found that there is highlighted strong associations between serum testosterone levels and disease progression and clinical outcomes in male COVID-19 patients [12].

Inhibin B serves as the primary regulator of FSH secretion detectable throughout life through its negative feedback effect on FSH secretion [13]. Inhibin B levels are higher in men with apparently normal fertility than in those with infertility and abnormal spermatogenesis [14]. TNF-α, IL-1, androgens, and epidermal growth factor have all been found as negative regulators of inhibin synthesis in Sertoli cells. When IL-1 is added to Sertoli cell cultures, activin A levels increase while inhibin B levels decrease [15]. The findings of Kazutaka and co-investigators [16] demonstrated the reciprocal link between FSH/cAMP signaling and inflammatory cytokine signaling pathways in the modulation of Sertoli cell activity, particularly inhibin B synthesis. These interactions are thought to be critical in the fine control of events during the seminiferous epithelium's cycle, as well as the inhibition of spermatogenesis during inflammation [16].

So far, no study has been found to connect the levels of inhibin B in adult males with the inflammatory mediators due to SARS-CoV2 infection. Investigation of the levels of testosterone and Inhibin B in adult males who are infected with SARS-CoV2 may help in understanding the mechanisms that affect their release in COVID-19 patients. This study attempts to explore and evaluate the correlation between the severity of COVID-19 and different contributors such as testosterone and inhibin B as sex-related parameters and the inflammatory mediators; IL-6 and TNF-a among Jordanian males.

## Materials and methods

### Study participants

This case-control study was performed on men aged 20-80 years between June and September 2021. All participants were divided into two groups; COVID-19 patients' group and healthy control group.

A total number of 88 men who visited the Precision Medical Lab with SARS-CoV-2 infection symptoms including fever, cough, headache, short breath, and fatigue were examined and diagnosed with SARS-CoV-2 infection by polymerase chain reaction assays (PCR) using nasopharyngeal swabs samples. Among them, 7 patients were excluded for any reason and the remaining 81 patients were included in the study for further research. Seventy-six healthy control individuals were recruited from the community and completed the study.

The participants of each group were further divided into two subgroups based on the age as follows: the first subgroup included patients in age between 20 and 50 years old and the second subgroup included patients with age 51-80 years old. Each subject was interviewed and completed a questionnaire. The biochemical tests that used in this study include testosterone, Inhibin B, TNF-α, and IL-6. The study's questionnaire and protocol were approved by the Institutional Review Board at Faculty of Allied Medical Sciences, Al-Ahliyya Amman University (IRB; AA-6-3-21) (Appendix 2). Additionally, informed written consent was taken from each participant prior to the commencement of the study to explain the benefits and/or any risk related to participation in the study.

### Methods

Almost 5 mL blood sample was collected into plain tubes from each participant collected between 8:00 am and 7:00 pm. Sera from blood samples were obtained by centrifugation at 4000 rpm for 7 minutes directly after clotting in the Precision Medical Lab and then transferred by an appropriate ice box to Pharmacological and Diagnostic Research Center (PDRC) at Al-Ahliyya Amman University, Amman, Jordan, where they have been stored at - 20 °C until analysis. Sandwich-ELISA methods were used for measurement of serum TNF-α, IL-6, and inhibin B following the manufacturer instructions (SUNLONG BIOTECH CO., China). The optical density (OD) was measured spectrophotometrically at a wavelength of 450 nm. The OD value is proportional to the concentration of TNF-α, IL-6, and inhibin B and the levels were calculated based on the standard curve. Testosterone levels were estimated using solid phase competitive ELISA kit (MyBioSource, San Diego, US). The optical density (OD) was measured spectrophotometrically at a wavelength of 450 nm. The intensity of color is inversely proportional to the concentration of testosterone in the samples. A standard curve was prepared to calculate the concentration of the testosterone. The absorbance (OD) results of ELISA plates were read using Biotek Microplate ELISA reader (Agilent, USA).

### Statistical analysis

Statistical analyses were performed using IBM SPSS statistical software version 22 (SPSS 22 Chicago, Illinois, USA). The data were expressed as means & standard deviations (SD). Comparisons of data between patients and healthy groups were made by independent Student's t-test. One-Way analysis of variance (ANOVA) with Bonferroni Post Hoc test was used to compare the differences between the age subgroups. Correlations analysis between different analyzed parameters was performed using Pearson's correlation coefficient test. The level of statistical significance of differences and correlations set at 5% (p < 0.05) was used. A significant level of *P* <0.05 was considered significant, and the level of *P* <0.01 was regarded as highly significant.

## Results

### Clinical characteristics of the study population

This study was performed on 157 adult males between the ages of 20-80 years who met the inclusion criteria and completed the questionnaire included in this study. The study participants consist of two groups; COVID-19 group (n = 81) and agematched healthy control group (n = 76). The clinical characteristics of the COVID-19 patients and healthy controls are summarized in Table 1. The mean value of age in the COVID-19 group was not statistically different from the healthy control group (54.35 ± 14.46 vs. 49.59 ± 15.80). The level of IL-6 was significantly higher in COVID-19 patients than healthy individuals (7.63 ± 6.30 vs. 5.54 ± 2.10, *P*=0.006). Similarly, the difference between the levels of TNF-α in the study groups were statistically significant (*P*=0.001) The mean testosterone levels in COVID-19 patients and healthy controls were 1.53 ± 1.24 and 3.87 ± 1.44, respectively, whereas the mean inhibin B levels in COVID-19 patients were 54.29 ± 7.33 as against 64.14 ± 37.66 in healthy controls, respectively. Testosterone and inhibin B levels are markedly lower (*P*<0.05) in COVID-19 patients than healthy controls.

**Table 1:** Laboratory characteristics of the study groups. IL-6, interleukin 6; TNF-α, tumor necrosis factor-alpha; SD, standard deviation

Parameter	COVID-19<br>Patients (n=81)		Healthy control<br>(n=76)		P value
	Mean	SD	Mean	SD	
Age (years)	54.35	14.46	49.59	15.80	0.051
IL-6 (ng/L)	7.63	6.30	5.54	2.10	0.006
TNF-α (ng/L)	24.58	10.97	19.24	8.45	0.001
Testosterone<br>(ng/mL)	1.53	1.24	3.87	1.44	< 0.001
Inhibin B<br>(ng/mL)	54.29	7.33	64.14	37.66	0.028

### Comparison of IL-6 and TNF-α between age groups in in the study participants

**Figure 1:**
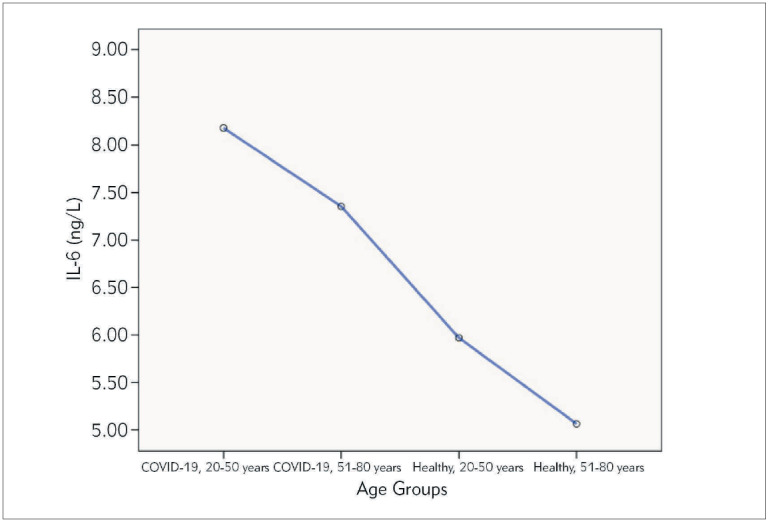
Levels of IL-6 in the different age groups of the study population.

Each study group was divided into 2 age subgroups; 20-50 years and 51-80 years as illustrated in Table 2. Levels of the inflammatory mediators and sex hormones were compared between COVID-19 patient and healthy control study groups according to the age subgroups. IL-6 and TNF-α levels were statistically different in the four age groups between COVID-19 patients and healthy controls; *P*=0.036 and *P*=<0.001, respectively (Table 2). It has been shown in Figure 1 a gradual decrease in the IL-6 levels starting from age 20-50 years COVID-19 patient followed by 51-80 years COVID-19 patients, 20-50 years healthy, and 51-80 years healthy controls. On the other hand, testosterone and inhibin B levels were significantly different in the four age subgroups of patients and healthy subjects (Table 2). Decreased levels of both testosterone and inhibin B were observed in COVID-19 patient compared to a healthy individuals with a more dramatic decreased testosterone levels (*P*< 0.001) as compared to inhibin B levels (*P*=0.011). The mean levels of TNF-α were higher in COVID-19 patients, in the age group 51-80 years old than the age group 20-50 years (Table 3), whereas IL-6, testosterone, and inhibin B levels were not significantly different between 20-50 years and 51-80 years subgroups.

**Table 2:** Levels of inflammatory mediators and sex hormones in the study groups based on age. ^*^, Based on ANOVA statistical analysis.

Parameter	COVID-19 Patients (n=81)		Healthy control (n=76)		P value^*^
	20–50 years<br>(n=27)	51–80 years<br>(n=54)	20–50 years<br>(n=40)	51–80 years<br>(n=36)	
IL-6 (ng/L)	8.18 ± 7.44	7.35 ± 5.71	5.97 ± 1.86	5.06 ± 2.27	0.036
TNF-α (ng/L)	20.88 ± 9.39	26.42 ± 11.31	17.93 ± 6.90	20.69 ± 9.78	< 0.001
Testosterone (ng/mL)	1.48 ± 1.22	1.56 ± 1.26	4.04 ± 1.51	3.67 ± 1.36	< 0.001
Inhibin B (ng/mL)	54.17 ± 7.34	54.35 ± 7.40	57.13 ± 27.58	71.93 ± 45.52	0.011

**Table 3:** The measured Laboratory parameters in different age groups of COVID-19 patients. NS, Non-significant difference.

Age subgroups	IL-6	TNF-α	Testosterone	Inhibin B
20–50 years (n=27)	8.18 ± 7.44	20.88 ± 9.39	1.48 ± 1.22	54.17 ± 7.34
51–80 years (n=54)	7.35 ± 5.71	26.42 ± 11.31	1.56 ± 1.26	54.35 ± 7.40
P value^*^	NS	0.023	NS	NS

**Figure 2:**
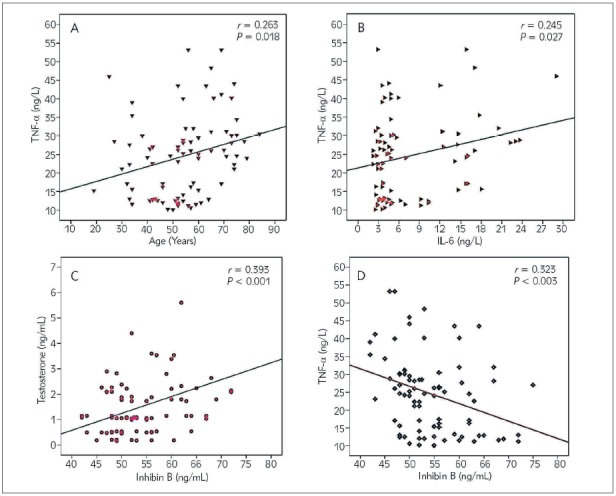
Pearson’s correlations between different variables. Positive correlations between TNF-α and age (A), TNF-α and IL-6 (B), and inhibin B and testosterone (C). Negative correlation between inhibin B and TNF-α (D) was observed.

Assessment of sex hormones levels correlation with age and inflammatory mediators in COVID-19 patients' group was shown in Figure 2. TNF-α exhibited significant positive correlation with age (*r* = 0.263, *P* = 0.018, Figure 2A) and IL-6 (*r* = 0.245, *P* = 0.027, Figure 2B). Although the correlation between TNF-α and testosterone was not significant (P>0.05), inhibin B had a significantly negative correlation (*r* = −0.326, *P* = 0.003, Figure 2C). Calculation of Pearson’s correlation coefficient showed that testosterone was positively correlated with inhibin B (*r* = 0.393, *P* < 0.001, Figure 2D). No significant correlations were observed between age and both inhibin B and testosterone levels. Inhibin B was estimated to be significantly correlated to TNF-α in the two age subgroups of COVID-19 patients but with different levels of significance as shown in Table 4. Inhibin B was significantly and inversely linked to TNF-α in the age group 20-50 years (r = -0.514, P = 0.006) and TNF-α in the age group 51-80 years (r = -0.270, P = 0.049). There was no significant correlation between testosterone and inflammatory mediators in both age subgroups.

**Table 4:** Correlation analysis between inflammatory mediators and sex hormones in the age subgroups of COVID-19 patients. r, Pearson’s correlation coefficient.

Parameter	20–50 years (n=27)				51–80 years (n=54)			
	IL-6		TNF-α		IL-6		TNF-α	
	*r*	*P*	*r*	*P*	*r*	*P*	*r*	*P*
Inhibin B	-0.249	0.210	-0.514	0.006	-0.030	0.827	-0.270	0.049
Testosterone	0.117	0.559	-0.187	0.349	-0.048	0.733	-0.131	0.344

## Discussion

Previous studies have reported that COVID-19 is one of the causative diseases for inflammation. Some evidence suggests that significant deterioration in some individuals during the coronavirus disease 2019 (COVID-19) epidemic was linked to excessive and dysregulated cytokine production [17]. TNF-α and IL-6 levels, as well as demographics, comorbidities, and other clinical and laboratory data, were shown to be significant in many previous studies. For example, the study by Del Valle el al. [18] is consistent with our results, which reported the higher levels of IL-6 and TNF-α in COVID-19 male patients than healthy subjects [18].

It has been suggested that testosterone controls several overlapping cellular and molecular processes involving a variety of immune cells and biochemical components, all of which come together to help reduce inflammation [19]. In the current study, it has been shown that testosterone levels were significantly lower in COVID-19 male patients compared to healthy controls, which is in parallel with the increased levels of TNF-α and IL-6 levels. SARS-CoV-2 infection status emerged to be independently linked with lower testosterone levels and these levels are suggestive of hypogonadism. In a study of Ma and coinvestigators [20] reduced testosterone and greater LH levels in a cohort of reproductive-aged SARS-CoV-2 infected males were observed when compared to age matched healthy controls.

On the other hand, the currents study shows that inhibin B levels are reported to be lower in COVID-19 patients than healthy controls and these results are comparable with the levels of testosterone that were decreased in COVID-19 patients. The decreased levels of inhibin B might be correlated with the men infertility. There have been no previous studies that suggest the effect of COVID-19 on the inhibin B levels and its consequences on the men infertility. However, recent investigations have documented the link of COVID-19 with the levels of testosterone levels [21].

In SARS-CoV-2 infected males, a sex-different sensitivity of the hypothalamic-pituitary-gonadal (HPG) to inflammation could cause an early and dramatic decline in circulating testosterone, and the resulting hypogonadal condition could lead to the fatal course of the disease [22]. Published evidence suggested that circulating testosterone levels may be negatively affected by unmodifiable variables (e.g., age), chronic problems (e.g., obesity, systemic disorders, and general health status), as well as acute sickness, including acute viral infections [23]. In our study, all COVID-19 cases were adjusted for the previous conditions as confounding factors by exclusion them from the study. It might be predicted that acute functional hypopituitarism, caused by a direct (virus) or indirect (cytokine storm) influence on the hypothalamus or pituitary gland, could result in acute testosterone depletion seen in patients with SARS-CoV-2 infection [24] as well as depletion of inhibin B in a similar mechanism.

Previous studies have investigated the variations of IL-6 and TNF-α levels in different age groups. For example, Milan-Mattos et al. [25] have reported the natural aging process could led to increased IL-6 and TNF levels, which is consistent with the inflaming theory; however, the 51-60 age range seems to be a key point for these increases. The current study findings revealed that IL-6 and TNF-α levels were statistically different in the four age groups between the study populations. It has been shown a gradual decrease in the IL-6 levels with higher levels in the age subgroup 20-50 years and 51-80 years of COVID-19 patient compared with the same age subgroups in the healthy controls. These findings demonstrated that age has a crucial role in the decreased concentrations of inflammatory markers in either age subgroup independent of the different cases in our study. However, no previous studies postulated the effect of age on the levels of inflammatory mediators including IL-6 and TNF-α in COVID-19 patients. Testosterone and inhibin B levels were significantly different in the four age subgroups of the study population. Xie and colleagues showed that although elderly persons have a greater mortality rate with COVID-19, they emphasized that men, regardless of age, have a higher mortality rate than women [26].

It has been recently reported that low testosterone levels predict clinical adverse outcomes in SARS-CoV-2 pneumonia patients but without a significant association between low testosterone levels and age [27] [28]. This is consistent with our results which revealed that, although testosterone and inhibin B levels were lower in COVID-19 patients than healthy controls, these hormones had no significant correlation with age, implying that low testosterone and inhibin B levels have an independent impact in causing poor outcomes in these patients.

In regard to the COVID-19, younger men routinely get better outcomes than elderly men. This is explained in our study by the significant positive correlation of TNF-α with IL-6 and age in COVID-19 patients. It is suggested that decreased testosterone and inhibin B levels in COVID-19 patients may indicate a hypogonadism. Our findings showed a significant and inverse connection between TNF-α and inhibin B and these findings are consistent with a previous study that reported hypogonadism to be associated with inflammatory markers [29]. This is the first study that shows the association of inhibin B with TNF-α in COVID-19 patients. The possible mechanism for the effect of TNF-α on the inhibin B levels might be related to the OS exerted on the Sertoli cells due to SARS-CoV-2 infection as OS produces oxidative damage to reproductive cells and intracellular components, which is harmful to male fertility indices. Other researchers observed that inflammatory mediators are chronically high in men whose testosterone levels are lower than the reference range [30].

It has been recently reported that oxidative stress (OS) is caused by an increase in the cytokine levels, which may lead to a systemic reaction by reducing testosterone hormone levels and affects the spermatozoa [31]. Production of IL-6 by the testes increases during inflammation in adult rats [32]. Our findings showed decreased levels of Inhibin B, but not testosterone, to be significantly and inversely linked to TNF-α levels in both age subgroups of COVID-19 patients. It is well known that when the testis is inflamed, the number of macrophages increases, and their function changes, as they release high levels of inflammatory cytokines including TNF-α and IL-6 [33].

Our findings suggest that most men seeking medical attention with symptomatic COVID-19 had low testosterone and inhibin B levels with increased both IL-6 and TNF-α, all of which might be linked to the worse clinical outcomes in these patients and conforming the deleterious effects of SARS-CoV-2 infection, which exerts effects on testicular function and immune response induction. However, IL-6 and TNF-α, but not testosterone and inhibin B are connected to older age in COVID-19 patients. These findings support our suggestion that lower testosterone and inhibin B levels, but not age, might be the cause of the more severe SARS-CoV-2 infections seen in our study population. Overall, our case-control study demonstrates that testosterone levels, inhibin B levels, and inflammatory markers in males with severe SARS-CoV-2 infections warrant clinical attention. Hence, the finding of this study may help to improve the current efforts of health organizations and providers about the effect of COVID-19 on test levels among male which can increase awareness among the community. These connections make the interactions of the inflammatory markers, specifically IL-6 and TNF-α with testosterone and inhibin B in COVID-19 patients an intriguing target of further exploration by immunologists, physiologists and drug researchers.

## Dodatak

### Acknowledgement

The authors are grateful for this financial support offered by Al-Ahliyya Amman University /Jordan. Corresponding author would like to thank International Institute Education – Scholar Rescue Fund (IIE-SRF) program for awarding the fellowship at Al-Ahliyya Amman University.

### Funding

This research was supported by Al-Ahliyya Amman University, Jordan.

### Author contribution statement

All authors contributed to the writing of the initial draft. Khaled Ahmed contributed to the discussion and edited the manuscript. Khalid Alqaisi reviewed the manuscript. Amneh Al-Bashiti did the practical work in the lab. All authors have read and agreed to the published version of the article.

### Ethical Approval

The study’s questionnaire and protocol were approved by the Institutional Review Board at Faculty of Allied Medical Sciences, Al-Ahliyya Amman University (IRB; AA-6-3-21).

### Conflict of interest statement

All the authors declare that they have no conflict of interest in this work.
